# Mechanical strength variability of deformed reinforcing steel bars for concrete structures in Ethiopia

**DOI:** 10.1038/s41598-022-06654-1

**Published:** 2022-02-16

**Authors:** Tariku Achamyeleh, Hüseyin Çamur, Mahmut A. Savaş, Ali Evcil

**Affiliations:** 1 Department of Mechanical Engineering, School of Applied Sciences, Near East University, 99138 Nicosia, KKTC Turkey; 2 Department of Mechanical Engineering, Faculty of Technology, Debre Tabor University, Debre Tabor, Ethiopia

**Keywords:** Engineering, Mathematics and computing

## Abstract

The paper presents statistical analysis of mechanical strength and linear density properties of deformed reinforcing Grade 60 steel bars. Two different lots of samples are identified based on test years for the years 2015–2017 as Lot 1 and 2018–2020 as Lot 2. Yield strength (YS), tensile strength (TS), elongation, mass per length and characteristic ratio of TS and YS are analyzed for rebar diameters of 8, 10, 12 and 16 mm considering both lots. Mechanical and linear density properties are compared statistically using range, mean, standard deviation, coefficient of variance, skewness and kurtosis of the recorded sets of values. Moreover, the results of YS, TS and elongation are analyzed separately by one-way analysis of variance for both lots. The result shows that the aggregate mean values of YS, TS and elongation for Lot 1 and Lot 2 are 593.1 MPa, 701.1 MPa, 14.78%; and 572.5 MPa, 673.8 MPa, 15.47%, respectively. Even though there is a slight decrement in values of YS and TS and increment in elongation from Lot 1 to Lot 2, both lots exceeded values recommended by ASTM A615 standard. Furthermore, with 95% confidence interval, one-way analysis of variance showed that the aggregate data of rebars are dissimilar in terms of YS, TS and percentage elongation with figures showing decrement from Lot 1 to Lot 2.

## Introduction

Concrete is resilient for compressive strength but weaker to tensile loads. Moreover, resistance of reinforced concrete structures for cyclic loading solely depends on the reinforcing steel bars which are considerably more demanding than its basic function. The energy absorption and dissipation during inelastic deformation depends almost entirely on the ductility of the reinforcing steel. Investigating the mechanical strength of reinforced steel bar determines the overall strength of concrete structure.

Reinforcing steel bars play a key role as a construction material. The properties of reinforcing steel bars must be known for the users before being applied for design or construction purposes because they are mainly used in construction projects that pose danger to human life such as building, bridges and furniture industries if improperly designed^1^.

Steel shows a wide range of mechanical and physical properties of which the mechanical strength factor is the dominant property^2^. The quality characteristics of reinforcing bars in steel structural engineering is commonly studied in terms of its mechanical properties such as, yield strength (YS), tensile strength (TS) and elongation^3^. These characteristics of steel are illustrated in generic stress–strain curve^1,4^. Thus, any improvement in strength characteristics of steel will promote the reliability and durability of the building structure in which it is used^2^.

Investigating mechanical properties of deformed reinforcement steel bars have become area of interest by many scholars particularly number of statistical studies conducted explicitly with the variability of the mechanical properties of reinforcing steel bars^2–18^. Effect of linear density properties and chemical composition variability studies were also investigated^19,20^.

In these studies, variability of yield strength, tensile strengths, elongation at fracture, mass per length and ratio of tensile strength to yield strength were examined and compared with ACI^21^, IS^22^ and ASTM standard^23–25^.

This variability is thought to be caused by dissimilarity in the manufacturing practices and quality control procedures followed by different firms, as well as possible variations in geometric size of the bars, steel strength, and rate of loading^6^.

According to Mirza and MacGregor^5^, test results of about 4000 ribbed reinforcing steel bars were studied to statistically determine relationships for various mechanical strengths. The study covered wide range of bars designated from No. 3 to No. 18. The sample included reinforcement steel bars of grade 40 and 60. The mean and coefficient of variation of the two grades were analyzed for yield strength and tensile strength.

Djavanroodi and Salman^6^ analyzed statistical variability of mechanical properties and weight of grade 60 reinforcing steel bars. The study investigated 130 samples for yield strength, tensile strength and elongation. 60 bars were investigated for variability of weight.

Carrillo et al.^4^ characterized stress–strain curves of steel reinforcing bars marketed in Bogota. 60 samples with diameters ranging from 9.5 mm to 25.5 mm were tested for tensile strength. The aggregate results were statistically analyzed and compared with other countries i.e. US, India and Mexico.

Tavio et al.^8^ in addition to yield strength, tensile strength and elongation, investigated ratio of tensile to yield strength of various steel grades and bar sizes. The test results were analyzed and compared among steel grades and with ASTM, IS^22^, ACI^21^ and Russian standards.

Rafi et al.^13^ investigated the variability of chemical and mechanical properties of cold-twisted and hot-rolled ribbed reinforcement steel bars of diameters ranging from 10 to 40 mm. The result showed a large variation in reinforcement bar strength for which design implications were studied as failure mode of flexure members might exhibit a change from ductile to brittle.

Bournonville et al.^26^ investigated statstical variability of mechanical properties of of reinforcement steel bars produced in the US and Canada. 100 samples were collected from 29 reinforcemnt steel manufacturing industries. The statistical report included parameters such as mean, median, standard deviation, cofficient of variance, skewness and kurtosis.

Alo et al.^27^ reported statistical assessment of mechanical properties of locally produced and imported reinforced steels used in Nigeria. One-way ANOVA analysis was conducted to indicate the variability of the reinforcing bars in terms of yield strength, tensile strength and elongation.

Variability analysis of mechanical properties of reinforcement steel bars plays a great role in assessing homogeneity of steel products used in the construction projects. Hence, the purpose of this work is to statistically analyze mechanical and linear density properties of reinforcing bars that were used in Ethiopian construction industry between the years 2015 and 2020 and make comparison with standards.

## Materials and methods

### Types and sample size of reinforcing steel

Two groups of samples are formed based on test period, namely Lot 1 and Lot 2 for tests conducted in the years 2015–2017 and 2018–2020, respectively. Both lots are collected from Ethiopian market irrespective of their source i.e. locally produced or imported steel bars. The sampling is carried out by the contractors and supervisors working in their respective construction projects. Samples are Grade 60 steel that comprise of four different diameters that are highly utilized by Ethiopian construction market^1^.

The sample size of each diameters and lots are listed in Table 1.

**Table 1:** Samples by lot and diameter.

Test sets	Diameter of bar (mm)				Total samples
	8	10	12	16	
Lot 1	37	44	40	31	152
Lot 2	39	27	60	51	177

### Test specimen preparation

All specimens were cut into a total length of 400 mm for tensile testing. The gauge length according to ASTM A615 is 200 mm. The remaining 200 mm is left for gripping at both ends.

### Tensile tests

The specimens are tested without any machining operation according to the provision by ASTM A615/A615M using universal testing machine (UTM) at Department of Mechanical Engineering Materials Testing Laboratory, Debre Tabor University. The UTM is a product of MATEST with a model of C140-09. The machine is capable of testing tensile, compression and three-point-bending specimens having different cross-sectional shapes. The jaws are separately made for different diameters ranging from 6 to 32 mm. The maximum possible applied force of the machine is 300KN which is calibrated for a load of 250KN with Load Cell 5000KN. The yielding strength, tensile strength and modulus of elasticity were recorded using UTM. The elongation of the steel samples was computed considering the original sample length and the final sample length at fracture. The tests performed are in triple sets and the average is used as presented in this data.

### Statistical analysis

Statistical means, standard deviations and ranges for variables of Lot 1 and Lot 2 are computed and presented. The variables are YS, TS, EL, mass per length and TS/YS.

Means and standard deviations are computed using the following procedure. After the desired numbers of test have been conducted on a particular lot for a specific variable, the mean value of *n* number of intercepts has been calculated according to:

1 $$ {\overline{\text{X}}} = \frac{{\sum {\text{X}}_{{\text{i}}} }}{{\text{n}}} $$

where *X*_*i*_ represents an individual value and *n* is the number of total tests.

The standard deviation (SD) of the individual measurements was calculated according to the equation:

2 $$ {\text{SD}} = \sqrt {\frac{{\sum ({\text{X}}_{{\text{i}}} - {\overline{\text{X}}})^{2} }}{{{\text{n}} - 1}}} $$

**Figure 1:**
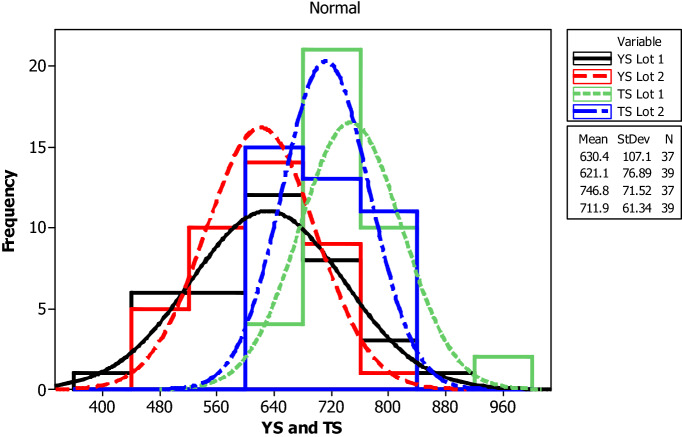
Histogram of YS and US of 8 mm diameter.

**Figure 2:**
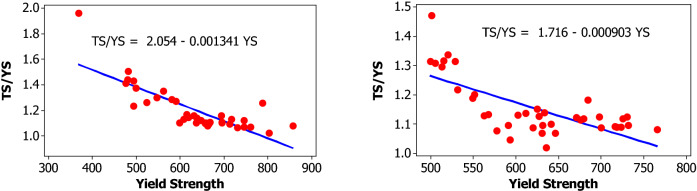
Fitted line plot (**a**) Lot 1 (**b**) Lot 2 for 8 mm diameter bar.

**Figure 3:**
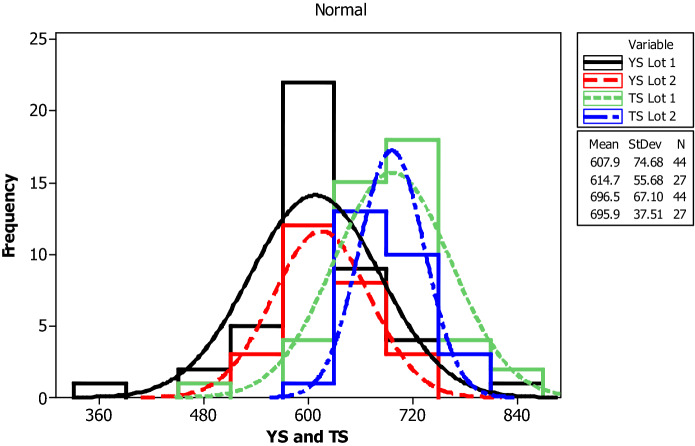
Histogram of YS and TS of 10 mm diameter.

**Figure 4:**
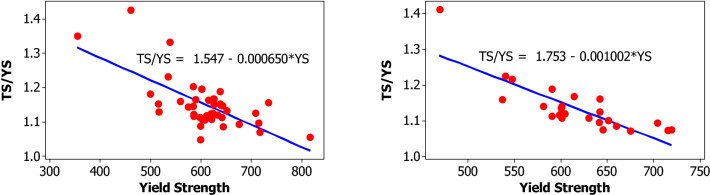
Fitted line plot (a) Lot 1 (**b**) Lot 2 for 10 mm diameter bar.

**Figure 5:**
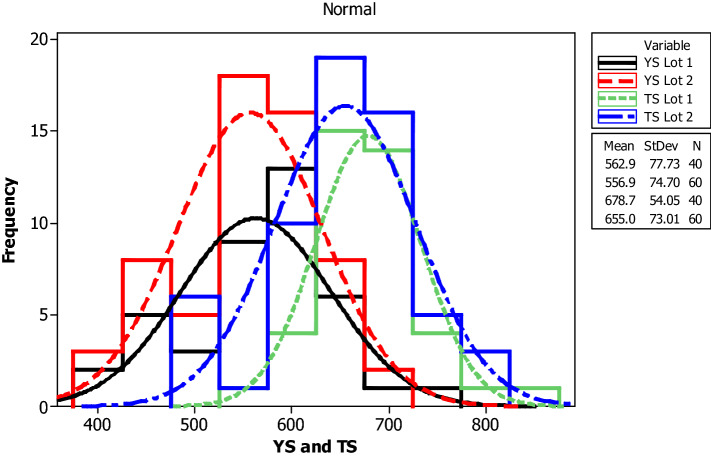
Histogram of YS and TS of 12 mm diameter.

**Figure 6:**
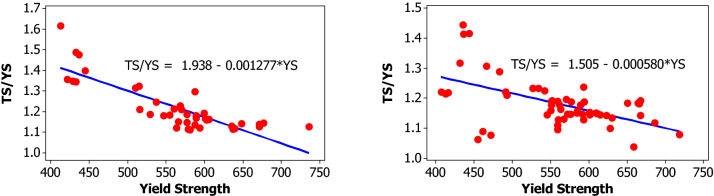
Fitted line plot (a) Lot 1 (b) Lot 2 for 12 mm diameter bar.

**Figure 7:**
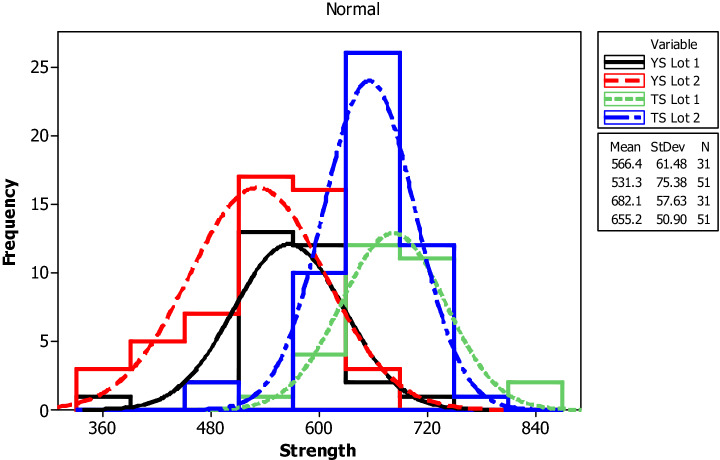
Histogram of YS and TS of 16 mm diameter.

**Figure 8:**
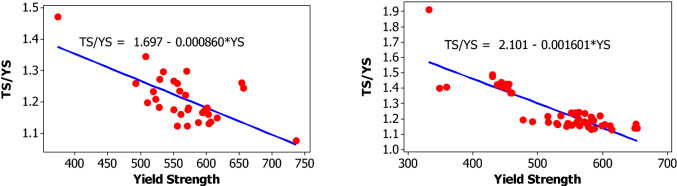
Fitted line plot (a) Lot 1 (b) Lot 2 for 16 mm diameter bar.

For yield strength, tensile strength, elongation percentage and mass per length, the following parameters are evaluated for each bar size in each lot: mean, median, standard deviation, coefficient of variation, minimum, maximum, skewness, and kurtosis. The skewness is a measurement of data symmetry. Negative values show data that is skewed to the left while positive values indicate data that is skewed to the right. The kurtosis is a degree whether the data is peaky or flat relative to a normal distribution. An increased kurtosis indicates an increased peak near the mean of the data. Ranges are determined as minimum and maximum values from the total test conducted (Figs. 1, 2, 3, 4, 5, 6, 7, 8). Summary statistics for bars of each size and lot are included in Tables 3, 4 and 6. Additionally, Figs. 1, 3, 5, and 7 depict histogram of data for yield and tensile strength values of all bars sizes in each lot. Regression models are also plotted to show the relationship between TS/YS and YS and presented in Figs. 2, 4, 6 and 8.

One-way analysis of variance for one factor one level test type is designed by which YS, TS, EL and TS/YS for the aggregate in each lot are analyzed using ANOVA^1,28^ as presented in Table 2.

**Table 2:** Generalized analysis of variance table.

Source of variation	Degree of freedom, f	Sum of squares, SS	Variance,	Variance ratio, F-cal	Pure sum of squares, Pure SS	Percent contribution, P
Mean (m)	f_m_	S_m_	V_m_ = S_m_/f_m_	V_m_/V_e_	S^’^_m_ = S_m_ – V_e_	S^’^_m_/S_T_
Errors (e)	f_e_	S_e_	V_e_ = S_e_/f_e_	–	S^’^_e_ = S_m_ + V_e_	S’_e_/S_T_
Total (T)	f_T_	S_T_				

f = Degree of freedom; n = Total number of results; f_T_ = Total degree of freedom = n – 1; Y_bar_ = Mean values of the results = $\sum\nolimits_{i = 1}^{n} {Y_{i} }$; S_T_ = Sum of squares of the total results = $\sum\nolimits_{i = 1}^{n} {(Y_{i} - Y_{0} )^{2} }$; S_m_ = Sum of squares of the mean = $n(\overline{Y} - Y_{0} )^{2}$; S_e_ = Sum of squares of the errors = S_T_ – S_m_ ; V_T_ = Variance of the total = S_T_ /f_T_; V_m_ = Variance of the mean = S_m_ /f_m_; V_e_ = Variance of the errors = S_e_ / f_e_; F_m_ = Variance ratio of the mean = V_m_ /V_e_; S^’^_m_ = Pure Sum of Squares for the mean = S_m_ – V_e_; S^’^_e_ = Pure Sum of Squares for the errors = S_m_ + V_e_

## Experimental results and discussion

This section discusses and summarizes the test results obtained in tensile test and linear density measurements (Tables 1, 2, 3, 4, 5, 6). Moreover, effect of yield strength to ratios of tensile and yield strength of the two lots are presented. The results of YS, TS, EL, M/L and TS/YS vs YS are compared one another for all bar sizes in both lots and aggregate results of each lot. All the mean values of YS, TS and EL were compared with ASTM A615/A615M recommended values of 420 MPa, 620 MPa and 9%, respectively. It’s also recommended that tensile strength should exceed yield strength by 25%. The recommended mass per length values are different based on bar diameters.

**Table 3:** Statistical analysis summary for 8 mm diameter bar.

Statistical Parameters	Lot 1				Lot 2			
	YS (MPa)	TS (MPa)	EL (%)	M/L	YS (MPa)	TS (MPa)	EL (%)	M/L
Min	368.00	609.67	7.20	0.3640	499.00	621.00	5.00	0.3600
Max	857.30	990.00	22.30	0.4550	766.00	828.00	21.50	0.4190
Range	489.30	380.33	15.10	0.0910	267.00	207.00	16.50	0.0590
Mean	630.37	746.85	13.57	0.3896	621.05	711.90	12.94	0.3817
Variance	11,470.67	5114.53	16.42	0.0003	5912.1	3762.1	15.9	0.0
Std. Dev	107.10	71.52	4.05	0.0171	76.89	61.34	3.99	0.01
COV	16.99%	9.58%	29.85%	4.38%	12.38%	8.62%	30.83%	3.15%
Skewness	−0.2264	1.3635	0.6419	1.7598	0.0005	0.4095	0.3135	0.4572
Kurtosis	−0.1346	3.2830	−0.2629	4.9964	−1.0999	−1.0876	−0.0432	1.1296

**Table 4:** Statistical analysis summary for aggregate.

Statistical parameters	Lot 1			Lot 2		
	YS (MPa)	TS (MPa)	EL (%)	YS (MPa)	TS (MPa)	EL (%)
Min	354.00	478.67	6.00	332.00	483.00	5.00
Max	857.30	990.00	28.20	766.00	828.00	27.00
Range	503.30	511.33	22.20	434.00	345.00	22.00
Mean	593.06	701.14	14.78	572.46	673.85	15.47
Variance	7427.64	4641.75	15.77	6591.53	4159.22	13.40
Std. Dev	86.18	68.13	3.97	81.19	64.49	3.66
COV (%)	14.53	9.72	26.87	14.18	9.57	23.66
Skewness	0.0164	0.6446	0.8608	−0.3090	−0.3457	0.3678
Kurtosis	0.8105	2.5876	1.5969	0.1415	1.1506	1.0520

**Table 5:** ANOVA table for aggregate data of Lots 1 and 2.

Source	Lot 1						Lot 2					
	DOF	SS	Variance	F-cal	Pure SS	P	DOF	SS	Variance	F-cal	Pure SS	P
Yield Strength												
Mean	1	2,691,059	2,691,059	362.30	2,683,632	0.70	1	2,238,469	2,238,469	339.60	2,231,877.53	0.66
Errors	151	1,121,574	7427.64	1	1,129,002	0.30	176	1,160,110	6591.53	1	1,166,701.47	0.34
Total	152	3,812,633	25,083.1	–			177	3,398,579	19,201	–		
Tensile Strength												
Mean	1	1,554,852	1,554,852	334.97	1,550,210	0.69	1	965,260	965,260	232.08	961,100.90	0.57
Errors	151	700,905	4641.75	1	705,546	0.31	176	732,023	4159.22	1	736,182.10	0.43
Total	152	2,255,756	14,840.5	–			177	1,697,283	9589.17	–		
Elongation Percentage												
Mean	1	5069.36	5069.36	321.52	5053.60	0.68	1	6366.30	6366.30	329.07	6346.96	0.65
Errors	151	2380.80	15.7669	1	2396.56	0.32	176	3404.95	19.35	1	3424.29	0.35
Total	152	7450.16	49.0142	–			177	9771.25	55.20	–		
TS/YS												
Mean	1	0.46	0.458	30.98	0.44	0.16	1	0.57	0.57	43.95	0.56	0.20
Errors	151	2.23	0.01478	1	2.25	0.84	176	2.29	0.01	1	2.30	0.80
Total	152	2.69	0.0177	–			177	2.86	0.02	–

**Table 6:** Statistical analysis summary for 10, 12 and 16 mm diameter bars.

Statistical parameters	Lot 1				Lot 2			
	YS (MPa)	TS (MPa)	EL (%)	M/L	YS (MPa)	TS (MPa)	EL (%)	M/L
	10 mm diameter bar							
Min	354.00	478.67	6.00	0.5740	469.00	623.00	11.50	0.5910
Max	816.67	862.67	24.50	0.7260	719.00	773.00	17.00	0.6163
Range	462.67	384.00	18.50	0.1520	250.00	150.00	5.50	0.0253
Mean	607.93	696.50	13.67	0.6114	614.74	695.85	14.35	0.6006
Variance	5576.43	4502.49	12.79	0.0009	3100.58	1407.05	2.69	0.0000
Std. Dev	74.68	67.10	3.58	0.0304	55.68	37.51	1.64	0.0059
COV (%)	12.28	9.63	26.16	4.97	9.06	5.39	11.43	0.98
Skewness	−0.4809	−0.3386	0.3560	2.2653	−0.2608	0.5926	0.0439	0.5785
Kurtosis	3.2606	2.3376	1.2777	5.9098	0.8875	−0.0384	−1.4550	0.3221
	12 mm diameter bar							
Min	412.30	570.30	10.67	0.7630	407.00	483.00	10.00	0.7730
Max	735.50	828.00	28.20	0.9470	718.00	794.00	25.00	0.8970
Range	323.20	257.70	17.53	0.1840	311.00	311.00	15.00	0.1240
Mean	562.86	678.70	15.93	0.8768	556.85	655.03	15.75	0.8581
Variance	6041.79	2921.08	17.21	0.0010	5579.96	5330.37	11.20	0.0008
Std. Dev	77.73	54.05	4.15	0.0318	74.70	73.01	3.35	0.0277
COV (%)	13.81	7.96	26.05	3.62	13.41	11.15	21.25	3.22
Skewness	−0.3492	0.3640	1.5455	−0.8728	−0.2348	−0.4480	0.8126	−1.1662
Kurtosis	−0.1277	0.6689	1.8970	3.6686	−0.4981	0.3993	0.5377	1.6154
	16 mm diameter bar							
Min	373.67	550.00	10.83	1.4900	332.00	487.00	12.00	1.1760
Max	736.67	825.67	27.80	1.6280	653.00	759.00	27.00	1.6300
Range	363.00	275.67	16.97	0.1380	321.00	272.00	15.00	0.4540
Mean	566.37	682.13	16.29	1.5541	531.27	655.24	17.68	1.5244
Variance	3779.91	3321.71	11.26	0.0011	5682.76	2590.90	9.71	0.0082
Std. Dev	61.48	57.63	3.36	0.0335	75.38	50.90	3.12	0.0908
COV (%)	10.86	8.45	20.60	2.16	14.19	7.77	17.63	5.96
Skewness	−0.2381	0.6323	1.7666	0.2582	−0.8405	−0.9174	1.2924	−3.2391
Kurtosis	3.5941	1.4930	4.7705	0.3374	0.3077	2.5054	1.9690	10.5545

### Mechanical and linear density properties for 8 mm diameter bars

It is found that the bars investigated exhibit variability in yield and tensile strengths. One (1) bar from a sample of thirty seven (37) bars (i.e. 2.70%) from Lot 1, exhibits a yield strength below the value specified by the ASTM A615/A615M standard while the remaining thirty six (36) bars have the mean yield strength greater than 420 MPa. In Lot 2, all the thirty nine (39) bars fulfilled the requirement of ASTM A615/A615M standard. Based on tensile strength, one bar (i.e. 2.70%) from Lot 1 exhibited tensile strength below the value specified by ASTM A615/A615M standard. But, all the bars in Lot 2 have a tensile strength greater than 620 MPa as per the requirement of ASTM A615/A615M.

The elongation percentage showed significant variability for 8 mm diameter bars in both lots. The mean elongation percentage in Lot 1 and Lot 2 are 13.6 and 12.9, respectively. There is no specified ASTM requirement for 8 mm diameter steel bar.

For 8 mm diameter reinforcing bar, the histogram and distribution curves of yield and tensile strengths are presented in Fig. 1. Lot 1 and Lot 2 showed significant variability for yield and tensile strength. The average yield strength of samples in Lot 1 and Lot 2 were 630.4 MPa and 621.1 MPa, respectively. The average tensile strength of samples in Lot 1 and Lot 2 were 746.8 MPa and 711.9 MPa, respectively. Moreover, Lot 1 had greater SD and COV values than Lot 2 in both yield and tensile strength results.

The influence of yield strength (YS) on the TS/YS ratio is found to be as expected for Lot 1 and Lot 2. The ratio decreases with increasing the yield strength which in turn is expected to reduce the ductility of the materials at higher yield strength. The mean TS/YS ratio of Lot 1 and Lot 2 is 1.20 and 1.15, respectively, which is below ASTM requirement^25^. 68% of the samples in Lot 1 and 82% in Lot 2 fail to meet the ASTM requirement for TS/YS ratio.

The statistical analysis including the values of maximum, minimum, average, variance, coefficient of variance, standard deviation, skewness, and kurtosis for yield strength, tensile strength, elongation and mass per length are calculated. Tables 3, 4 and 6 summarize the statistical analysis of yield strength, tensile strength, elongation and mass per length for 8, 10, 12, and 16 mm diameter bars and aggregate result of the two lots. From the results of COV, it’s noted that mass per length in both lots show high homogeneity (COV < 5%)^4^ while the rest of the data show moderate homogeneity.

### Mechanical and linear density properties for 10 mm diameter bars

One (1) bar from a sample of forty four (44) bars (i.e. 2.27%) from Lot 1, exhibits yield strength below the value specified by the ASTM A615/A615M standard while in Lot 2, all the twenty seven (27) bars have the yield strength greater than 420 MPa as per the requirement of ASTM. Based on tensile strength, four bars (i.e. 9.09%) from Lot 1 exhibited tensile strength below the value specified by ASTM A615/A615M standard. But, all the bars in Lot 2 have tensile strength greater than 620 MPa as per the requirement of ASTM A615/A615M.

Five bars (i.e. 11.36%) in Lot 1 exhibits elongation percentage lower than ASTM A615/A615M requirement while all bars in Lot 2 surpasses the minimum elongation percentage requirement. The mean elongation percentages in Lot 1 and Lot 2 are 13.7 and 14.4, respectively.

In mass per length analysis, 5 bars (i.e. 11.36%) in Lot 1 fail to meet the minimum ASTM standard for their bar size and grade. But, all the bars in Lot 2 meet the minimum requirement. The mean mass per length values in Lot 1 and Lot 2 are 0.6114 and 0.6001, respectively.

For 10 mm diameter reinforcing bar, the histogram and distribution curves are presented in Fig. 3. Lot 1 and Lot 2 show significant variability for yield and tensile strength. The mean yield strength of samples in Lot 1 and Lot 2 are 607.9 MPa and 614.7 MPa, respectively. The mean tensile strength of samples in Lot 1 and Lot 2 are 696.5 MPa and 695.9 MPa, respectively.

Even though the ratios decreased with increasing the yield strength which in turn is expected to reduce the ductility of the materials at higher yield strength, the mean TS/YS ratio of Lot 1 and Lot 2 was 1.15 and 1.13, respectively, which was below ASTM requirement. 91% of the samples in Lot 1 and 96% in Lot 2 fail to meet the TS/YS ratio of ASTM requirement. Figure 4 shows the linear regression line plot of TS/YS to YS for both lots.

### Mechanical and linear density properties for 12 mm diameter bars

One bar from a sample of 40 (i.e. 2.50%) bars in Lot 1 exhibits a yield strength which is below the value specified by the ASTM A615/A615M standard whereas in Lot 2, 3 bars from a sample of 60 ( i.e. 5.00% ) bars fail to meet the minimum requirement for yield strength set by ASTM standard. Based on tensile strength, 3 bars (i.e. 7.5%) in Lot 1 and 14 (i.e. 23.33%) in Lot 2 exhibit tensile strength below the value specified by ASTM A615/A615M standard.

The average yield strength of samples in Lot 1 and Lot 2 are 562.9 MPa and 556.9 MPa, respectively. The average tensile strength of samples in Lot 1 and Lot 2 are 678.7 MPa and 655.0 MPa, respectively. For 12 mm diameter reinforcing bar, the histogram and distribution curves are presented in Fig. 5.

All samples in both lots exhibit elongation percentage values greater than the minimum requirement set by ASTM A615/A615M standard. The mean elongation percentages are 15.9 and 15.8 in Lot 1 and Lot 2, respectively.

In mass per length analysis, all bars in both lots meet the minimum ASTM standard. The mean mass per length values in Lot 1 and Lot 2 are 0.8768 and 0.8581, respectively.

The TS/YS ratios show decrement with increasing the yield strength. The mean TS/YS ratio of Lot 1 and Lot 2 was 1.22 and 1.18, respectively, which is below ASTM requirement^25^. 75% of the samples in Lot 1 and 90% in Lot 2 fail to meet the TS/YS requirement set by ASTM.

### Mechanical and linear density properties for 16 mm diameter bars

One (1) bar from a sample of thirty one (31) bars ( i.e. 3.23% ) in Lot 1 and three (3) bars from a sample of fifty one (51) (i.e. 5.88%) in Lot 2 exhibited a yield strength below the value specified by the ASTM A615/A615M standard. Based on tensile strength, two bars (i.e. 6.45%) in Lot 1 and six bars (i.e. 11.76%) in Lot 2 exhibited tensile strength below the value specified by ASTM A615/A615M standard. The mean yield strength of samples in Lot 1 and Lot 2 were 566.4 MPa and 531.3 MPa, respectively. The mean tensile strength of samples in Lot 1 and Lot 2 were 682.1 MPa and 655.2 MPa, respectively. For 10 mm diameter reinforcing bar, the histogram and distribution curves are presented in Fig. 7.

The mean elongation percentages are 16.3 and 17.7 in Lot 1 and Lot 2, respectively and all bars in both lots surpass the minimum elongation percentage requirement set by ASTM.

The mean mass per length values in Lot 1 and Lot 2 are 1.5541 and 1.5240, respectively and all bars in both lots surpass the minimum requirement.

The TS/YS ratios showed decrement with increasing the yield strength. The mean TS/YS ratio of Lot 1 and Lot 2 was 1.21 and 1.25, respectively. 71% of the samples in Lot 1 and 75% in Lot 2 failed to meet the minimum TS/YS requirement i.e. 1.25 set by ASTM.

### Mechanical strength variability of aggregate results

**Figure 9:**
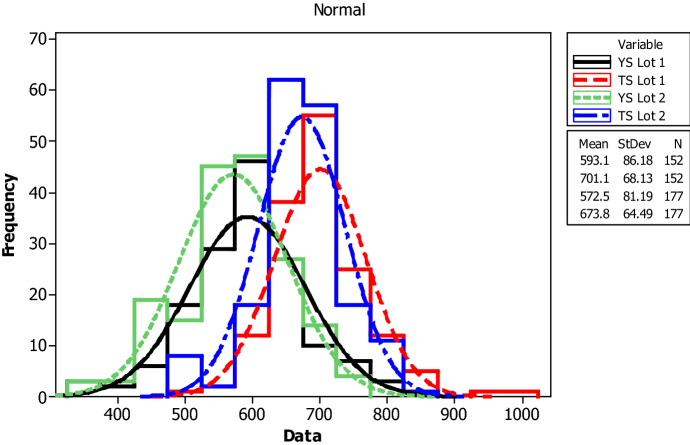
Histogram of YS and TS of aggregate.

Figure 9 and Table 4 summarize the aggregate results of yield strength, tensile strength and elongation percentage of the two lots irrespective of diameter of the bars. From the aggregate result, it is noted that the parameters in both lots show moderate homogeneity. The mean yield strength value shows a decrease from 593.1 MPa in Lot 1 to 572.5 MPa in Lot 2. Similarly, the ultimate strength decreases from 701.1 MPa in Lot 1 to 673.8 MPa in Lot 2. Even though the aggregate result shows a decrease in overall mechanical strength values from Lot 1 to Lot 2, the mean yield and tensile strength values are higher than the findings from others studies^3,4,6,7,14,15,29,30^.

The mean elongation percentage, on the contrary, shows an increase from 14.78 in Lot 1 to 15.47 in Lot 2. This result is close to the findings from few studies^4,6^ but less than the findings from other studies^3,13,29^.

The skewness values of the strength parameters of the aggregate result exhibits a positive value in Lot 1 where as in Lot 2 yield strength and tensile strength values have a negative skewness. Similarly, Lot 1 shows relatively higher kurtosis value than Lot 2.

ANOVA generalized table^1^ is used to compare calculated variance ratio (F-cal) with tabulated critical variance ratio (F-table) values for statistical significance value of 95%. This ratio was used to measure the significance of the factor under the investigation with respect to the variance of all other unseen factors. A factor is significant when F-cal is greater that F-table. Table 5 indicated the ANOVA results of Lots 1 and 2 in terms of YS, TS, EL and TS/YS.

From the ANOVA of yield stress in Lot 1, the computed value for F-cal value, 362.3 is greater than the critical F-table value for *f*_*. 05*_ (1, 152), 95% confidence i.e., 3.8415^1,28^. Hence, with 95% confidence, the reinforced bars appeared to be dissimilar. The apparent data spread contributes about 70% to the sample variability while the remaining 30% variation was caused by other factors. F-cal values of all parameters in both lots are greater than *f*_*. 05*_, 95% confidence for its respective tabular values. Percent contribution to the variability of the data in Lot 2 is less than in Lot 1 in terms of YS, TS and elongation percentages.

## Conclusion

This paper presented the results of a statistical analysis of all the relevant reinforcing steel properties of interest in Ethiopian structural construction. Over 300 data sets grouped in to two lots based on timeline of test were considered. The considered data sets include reinforcing steel bar sizes ranging from 8 to 16 mm that are widely used in different structural works in Ethiopia. Two lots that are representative of two separate test periods were compared one another based on mechanical strength and linear density property results. The comparison of these values in both lots showed that there was slight quality decline based on timeline of the tests. Aggregate strength values of YS and TS exhibited a decrease from 593.1 MPa in Lot 1 to 572.5 MPa in Lot 2 and from 701.1 MPa in Lot 1 to 673.8 MPa in Lot 2, respectively. Moreover, the results from the investigation showed that most of the samples in both lots have yield strength, tensile strength, elongation percentage and mass per length values that surpass the recommended requirements by ASTM. The result also showed that few samples had tensile to yield strength ratio that surpasses the recommended value by ASTM. The ANOVA analyses show that the data for YS, TS and TS/YS are dissimilar. In general, this class of steels used in the samples can be used for general structural applications but will not be acceptable in seismic-prone regions due to poor post elastic behavior. Material composition, source of the steel, corrosion and their effect on monotonic and fatigue loading behaviors of ribbed reinforcing Grade 60 steel bars should be studied further in the future.
